# The influence of weight excess on the postprandial lipemia in adolescents

**DOI:** 10.1186/1476-511X-12-17

**Published:** 2013-02-13

**Authors:** Viviane Sahade, Silvana França, Luis F Adan

**Affiliations:** 1 Department of Nutrition Science, Federal University of Bahia School of Nutrition, Bahia, Brazil; 2 Department of Pediatrics, Federal University of Bahia School of Medicine, Bahia, Brazil; 3 State of Bahia Center for Diabetes and Endocrinology (CEDEBA), Bahia, Brazil

**Keywords:** Postprandial Lipemia, Adolescents, Overweight

## Abstract

**Background:**

Postprandial lipemia (PL) in adults has been extensively studied, but little explored in youth. The aim of this study was to evaluate the influence of weight excess on postprandial lipemia in adolescents.

**Methods:**

Eighty-three adolescents were classified into Groups 1 (n= 49, overweight) and 2 (n=34, eutrophic). Total cholesterol (TC), triglycerides (TG), HDL and LDL cholesterol were measured before, 2 and 4 hours after a standardized 25 g lipid and 25 g of carbohydrate test meal; glycemia and insulin measured only at baseline. Anthropometric evaluation was performed.

**Results:**

Basal TG were higher in Group 1 (p= 0.022). The total increase (Δ-TG), corresponding to the difference between the maximum and the basal TG level was similar in both groups (29.8 ± 21.5 mg/dl *vs*. 28.2 ± 24.5 mg/dl, p= 0.762). TC, HDL and LDL did not change significantly throughout the test. By analyzing all the adolescents together, the waist circumference was positively correlated with TG at fasting (r = 0.223; p= 0.044) and at 4 hours (r = 0.261; p= 0.019). Only overweight adolescents with hypertriglyceridemia, who also had higher HOMA-IR, presented significant elevation of TG levels 2 and 4 hours after the overload.

**Conclusion:**

The behavior of lipoproteins in the post-prandial state is similar in eutrophic and overweight adolescents. Thus, apparently the weight excess does not induce post prandial lipemic alterations.

## Background

The fasting state occurs, by definition, after an 8-hour fast; thus, most humans find themselves in the nonfasting state for the larger portion of a 24-hour period, perhaps with the exception of the early morning hours. Despite this fact, plasma lipids, lipoproteins, and apolipoproteins for cardiovascular risk prediction are usually measured in the fasting state [1].

Postprandial lipemia is a metabolic condition related to an increase in plasma triglycerides (TG) [2]. Due to high-fat diets and frequent feeding, TG levels may be elevated all day long, exposing individuals to large quantities of atherogenic TG-rich lipoproteins that can penetrate and reside in the subendothelial space, contributing to foam cell formation and promoting lipid accumulation in the vessel wall [3]. Postprandial lipemia responses are elevated in peripheral artery disease [4], hypertension [5] and coronary heart disease (CHD) [6]. Thus, atherogenesis can be considered a postprandial phenomenon [7].

It is not uncommon for adolescents in industrialized nations to spend considerable time in the postprandial state in a typical day and there is now compelling evidence that these atherosclerotic processes begin early in childhood, and are influenced over the course of life by genetic and potentially modifiable risk factors [8].

The purpose of this study was to evaluate the influence of overweight in adolescents in postprandial lipemia.

## Materials and methods

### Sample size

The minimum sample size calculation was performed using the statistical program WINPEPI, assuming an alpha of 5% and power of 80%. The ratio number of eutrophic adolescents to adolescent with excess weight was 1.0, with standard deviations the triglycerides of 33.9 mg/dL and 13.2 mg/dL respectively for the eutrophic and overweight adolescents. The expected difference between the mean increase of TG at 0 and at 2 hours was 15 mg/dL. These data was obtained from the study itself when the sample achieved 34 and 49 adolescents respectively in the eutrophic and in the overweight groups.

### Subjects

The sample was composed by 49 consecutive overweight adolescents selected between July 2009 and February 2011, through an outpatient pediatric endocrinology unit (State of Bahia Center for Diabetes and Endocrinology - CEDEBA); the 34 eutrophic adolescents came forward after seeing advertisements inviting volunteers.

The subjects were classified into 2 groups based on the body mass index: Group 1, overweight adolescents (body mass index ≥ 85th percentile); Group 2, eutrophic (body mass index between the 3rd and the 85th percentile) [9].

Subjects were excluded if they had diabetes, thyroid dysfunction, kidney disease, chronic inflammatory diseases, history of recent intercurrent disease, were pregnant or lactating, or if they were taking any medications or dietary supplements.

### Body composition and clinical examination

Measurements of height, weight, waist circumference (WC) and skin fold thickness were obtained from the adolescents. We used mechanical balance Welmy up to 150 kg, stadiometer fixed to wall, non-elastic tape measure millimeter and Lange´s plicometer. All measurements were made in triplicate, using the arithmetic mean as the final measure.

This anthropometric evaluation was obtained by the same nutritionist.

Height measurement was taken without shoes. The adolescents were positioned with their feet together and flat on the base plate with their head and back straight against the vertical measuring rods. Once the correct position was achieved the interviewer lowered the head plate until it just touched the top of the adolescent's head, and while maintaining this position, he or she was asked to stand as tall as possible, without lifting the heels. Measurements were made to the nearest 0.1 cm. Weight measurement was taken in light clothing; shoes, jackets, heavy jewelry, keys and wallets were removed. Weight was recorded to the nearest 0.1 kg.

Body mass index (BMI) was calculated as weight (kg) divided by height (m) squared and then compared to the newly recommended NCHS/WHO reference standards [9]. Subcutaneous fat was estimated by measuring triceps (TSF) and subscapular (SSF) skinfold thickness (mean of three readings), on the left side of the body, using Lange calipers (Cambridge Scientific Industries, Inc, Cambridge, MD).

Measurement of TSF was taken on the posterior aspect of the bare extended left arm, over the triceps muscle, midway between the lateral projection of the acromion process of the scapula and the inferior margin of the olecranon process of the ulna. The caliper tips were placed perpendicular to the long axis of the skin fold, and the reading on the dial was taken to the nearest 0.1 mm. SSF was measured 2 cm below the lowest or inferior angle of the scapula. The long axis of the skin fold was at a 45° angle directed to the right side. With the adolescent's arms relaxed to the sides, the skin was grasped 1 cm above and medial to the site along the axis. A measurement was taken to the nearest 0.1 mm.

To measure the WC, an inelastic tape was applied horizontally midway between the lowest rib margin and the iliac crest about the level of the umbilicus. Central obesity was defined as WC > 75th percentile for age and gender, as proposed by Fernandez et al [10].

### Oral fat load test

A pilot study including 38 adolescents was conducted in order to determine the adequate carbohydrate and lipid concentrations in the drink proposed for the protocol. The subjects tasted two drinks with different concentrations: drinks A (50 g of carbohydrate and 100 ml of fat) and B (25 g of carbohydrate and 50 ml of fat); the taste and texture of drink B were better accepted by the group. Due to the results obtained from the pilot study, a 100-ml test drink was formulated by adding 25 g of carbohydrate (spray-dried maltodextrin gluten-free; Support Nutritional Products) to 50 ml of a commercially produced strawberry-flavored fat emulsion containing 25 g long-chain triglycerides (Calogen; Support Nutritional Products). Nutritive constituents of the test drink, which had an energy content of 1.357,5 kJ (328 kcal), are listed in Table 1.

**Table 1:** Test drink nutrient composition

**Component**	**Per 100 ml**
Fat (g)	25.0
Saturated (%)	10.7
Monosaturated (%)	60,7
Polyunsaturated (%)	28,6
Carbohydrate (g)	25 g
Glucose (%)	2.8
Maltose (%)	5.6
Polysaccharides (%)	91.6
Protein (g)	0.0 g
Energy (kJ/ kcal)	1357,5 / 328

The 100 ml test drink was formulated by adding 25 g of carbohydrate (spray-dried maltodextrin gluten-free; Support Nutritional Products) to 50 ml of a commercially produced strawberryflavored fat emulsion (Calogen; Support Nutritional Products), containing 25 long-chain triglyceride.

Considering that fat is usually ingested with carbohydrate, the incorporation of 25 g of maltodextrin in the drink makes it more akin to everyday meals and thus more likely to reflect the daily postprandial situation than an isolated fat load.

Subjects were asked not to consume alcohol or undertake any undue exercise 24 h before the load test. Blood samples were obtained at time intervals of 0, 2 and 4 hours after ingestion. In each sample, total cholesterol (TC), triglycerides (TG), HDL cholesterol and LDL cholesterol were determined. Insulin and glucose were collected only at time 0.

Subjects were admitted early in the morning (7:00 a.m.) after a 12-h overnight fast (water permitted). A 20-G venous cannula was inserted into a forearm vein, and blood samples were collected for measurement of fasting plasma lipid fractions, insulin and glucose levels. The test drink was served chilled for ingestion within 2 - 3 min. During the test, subjects were allowed to drink water, but no food intake or smoking. They could move around freely but were asked no to perform any type of physical activity. Venous blood samples were drawn at time intervals of 2 and 4 h for determination of lipid levels.

During the following four hours the adolescents participated in an educational program that included discussion on obesity prevention and treatment, dyslipidemia and cardiovascular diseases.

### Biochemical and hormonal assays

Blood samples were centrifuged at 3,500 rpm for 10 min and the plasma was separated. Glucose, total cholesterol and triglycerides were measured by the enzymatic method with Wiener reagents, and HDL cholesterol by means of Labtest Direct-HDL reagents, all of them in 3000 BT Equipment Plus. Insulin was measured by chemiluminescent assays in Siemens equipment Immulite 2000. The LDL-cholesterol values were calculated according to Friedewald et al [11].

The absolute TG increase at the hour (Δ-TG) was taken as the difference between the maximum triglyceride levels and baseline. Insulin resistance was estimated by the homeostasis model assessment (HOMA-IR = [fasting glucose × fasting insulin] / 22.5) and the cutoff point used to was 3.16 [12].

### Statistical analysis

Data are given as means ± standard deviation (SD) for normally distributed variables, and otherwise as medians (25th and 75th percentile values). The normal distribution of the variables was evaluated by the Kolmogorov-Smirnov test. Mean values and median value were compared between overweight and nonobese adolescents using unpaired Student’s *t*-test and Mann-Whitney *U*-test as appropriate. For dependent groups the t test or the Wilcoxon sign rank test was used. For comparisons between them ANOVA or Kruskal-Wallis test was performed.

In a secondary analysis, Group 1 (overweight) was subdivided into 1a (fasting TG < 100 mg/dl) and 1b (fasting TG ≥100 mg/dl); and Group 2 (normal weight) into 2a (fasting TG < 100 mg/dl) and 2b (fasting TG ≥100 mg/dl)

Differences in metabolic parameters, incremental triglyceride response and HOMA-IR between the four groups were examined by one-way ANOVA. Post hoc analyses were used when indicated. Pearson’s correlation coefficient was used to determine the correlation between continuous parameters. An alpha level ≤ 0.05 was accepted as significant for all statistical procedures. All statistical analyses were conducted using SPSS for Windows software version 17.0 (SPSS Inc., Chicago, IL, USA).

### Ethical aspects

The nature and purpose of the study were carefully explained to both parents and adolescents before obtaining written voluntary consent to participate in the study. The protocol was approved by the CEDEBA Human Research Ethics Committee.

## Results

### Subjects at baseline

A total of 83 adolescents were included, 49 with overweight (Group 1) and 34 eutrophic (Group 2). The test drink was well accepted by all adolescents, with no episode of nausea, vomiting or abdominal discomfort after consumption.

The demographic, anthropometric and laboratory data are summarized in Table 2. The overweight adolescents had significantly higher amounts of body fat (expressed as higher BMI, waist circumference and triceps and subscapular skinfold thickness), insulin and HOMA-IR than eutrophic adolescents. Nine adolescents (7 overweight and 2 eutrophic) presented insulin resistance (HOMA-IR > 3,16).

**Table 2:** Demographic, clinical and laboratory data from 83 overweight and eutrophic adolescents

	**Overweight (n = 49)**	**Eutrophic (n = 34)**	**p**
Sex (M/F)	20 / 29	17 / 17	NS
Age, yr	12.0 (11.0; 14.0)	13.0 (11.0; 15.3)	NS
BMI (kg/m^2^)	29.1 (26.8; 32.6)	17.2 (16.1; 19.6)	<0.001
WC (cm)	95.6 ± 12.2	66.9 ± 8.0	<0.001
Σ TSF e SSF (mm)	55.5 (45.0; 61.5)	15.5 (13.0; 21.5)	<0.001
Glucose (mg/dl)	83.9 ± 5.2	84.2 ± 7.6	NS
Triglyceride (mg/dl)	102.5 ± 53.4	79.5 ± 36.4	0.022
Total cholesterol (mg/dl)	169.3 ± 37.4	162.8 ± 23.9	NS
HDL-c (mg/dl)	43.5 (36.9; 53.4)	47.9 (40.8;56.4)	NS
LDL-c (mg/dl)	98.1 (80.2;117.4)	94.6 (81.9; 114.0)	NS
Insulin (mU/ml)	9.2 (7.2; 14.8)	3.9 (2.8; 6.5)	<0.001
HOMA-IR	1.9 (1.5; 2.9)	0.8 (0.6; 1.4)	<0.001

NS, not significant, median (25th - 75th percentiles), mean ± SD.

### Basal lipid status and lipid concentrations after the oral fat load

Mean levels of fasting TG in Group 1 were higher than those in Group 2 (p = 0.022). The total increase (Δ-TG), corresponding to the difference between the maximum and the basal TG level was similar in both groups (29.8 ± 21.5 *vs*. 28.2 ± 24.5, p= 0.762). The levels of TC, HDL and LDL did not change significantly after the fat overload in both groups (Table 3).

**Table 3:** Fasting and postprandial triglyceride in 83 overweight and eutrophic adolescents

**Variables**	**Overweight (n =49)**	**Eutrophic (n=34)**	**p**
0 h	102.5 ± 53.4	79.5 ± 36.4	0.022
2 h	123.3 ±61.7	100.5 ± 53.0	0.084
4 h	126.2 ± 65.8	92.2 ± 40.5	0.005
p*=	0.114	0.143	
Δ -TG (mg/dl)	29.8 ± 21.5	28.2 ± 24.5	0.762
Δ - 2TG (mg/dl)	20.8 ± 17.9	21.1 ± 28.0	0.966
Δ - 4TG (mg/dl)	21.1 ± 30.0	12.7 ± 22.7	0.171

* p = Anova test.

When the sample was classified according to the presence of central obesity, the adolescents with waist circumference ≥ 75^th^ percentile presented higher mean insulin (10.9 mU/ml ± 6.5 *vs*. 5.8 mU/ml ± 3.8, p < 0.001) and HOMA-IR (2.2 ± 1.4 *vs*. 1.1 ± 0.8, p < 0.001) than those with waist circumference < 75^th^ percentile. Table 4 shows fasting and stimulated TG levels according to central obesity. Significant differences between the groups were found only at the 4^th^ hour after overload (p=0.020). However the increase in TG concentrations was similar in both groups (p=0.102) .

**Table 4:** Basal and stimulated TG levels in 83 adolescents according to central obesity

	**Central Obesity**		**p**
	**No (<P75)**	**Yes (≥P75)**	
	**(n = 33)**	**(n = 50)**	
BMI (kg/m^2^)	17.2 (16.0; 20.1)	29.0 (26.6; 32.6)	<0.001
0 h	81.9 ± 37.1	97.8 ± 51.2	0.133
2 h	105.6 ± 52.7	117.5 ± 61.9	0.372
4 h	94.1 ± 42.1	122.5 ± 65.4	0.020
Δ -TG (mg/dl)	28.9 ± 24.8	29. 8 ± 21.2	0.861
Δ - 2TG (mg/dl)	23.7 ± 27.5	19.7 ± 18.5	0.433
Δ - 4TG (mg/dl)	12.2 ± 23.1	22.2 ± 28.7	0.102

* p = Anova test.

In the entire group, the waist circumference was positively correlated with triglycerides in the fasting state (r = 0.223; p = 0.044) and at 4 hours (r = 0.261; p = 0.019). WC did not correlate with other lipid fractions.

### Triglyceridemia in overweight and eutrophic groups

The fasting TG levels showed great variation in the adolescents with and without excess weight, and for this reason, in a second stage, the sample was divided into 4 sub-groups: 1a, excess weight and fasting TG < 100 mg/dl; 1b, excess weight and fasting TG ≥ 100 mg/dl; 2a, eutrophic and fasting TG < 100 mg/dl; 2b, eutrophic and fasting TG ≥100 mg/dl.

As expected, there was difference in the fasting TG and post-prandial TG means between Groups 1a and 1b (p<0.001), as well as between Groups 2a and 2b (p<0.001).

The adolescents in Group 1b presented higher HOMA-IR (2.8 [1.8;3.1]) than those of groups: 1a (1.7 [0.97;2.25]; p = 0.025), 2a ( 0.7 [0.6;1.3]; p < 0.001) and 2b (0.9 [0.4;2.0]; p = 0.005). There was no statistically significant difference in the HOMA-IR when the other groups were compared among them. Similarly, there was no statistically significant difference in the base and post-prandial levels of TC, HDL and LDL in the four sub-groups analyzed.

**Figure 1:**
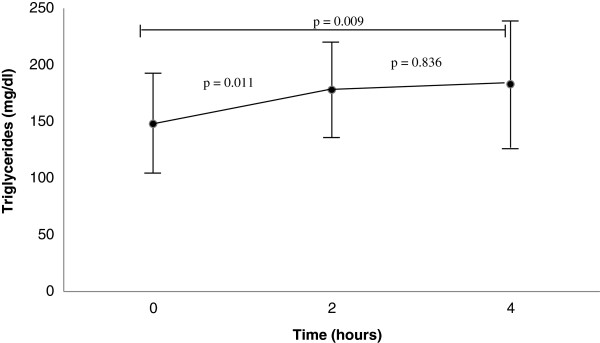
Fasting and post-prandial triglycerides levels in overweight adolescents with fasting hypertriglyceridemia

Group 1b was the only one that presented significant elevation in the fasting TG levels for the time intervals of 2 hours and 4 hours after ingestion of the shake (Figure 1).

## Discussion

In the present study it was observed that after the lipid overload test, the behavior of lipoproteins was similar in the adolescents with excess weight and eutrophic adolescents. In agreement with our findings, Moreno *et al.*[13] evaluated postprandial lipemia in adolescents (12 obese and 12 non-obese, aged 11.0 to 13.8 years) and also found no significant differences between the groups. Tiret *et al.*[14] in the European Atherosclerosis Research Study (EARS) compared the postprandial TG response of offspring whose father had suffered a myocardial infarction before the age of 55 years with controls in different populations in Europe. They found no differences between cases and controls in the postprandial TG response. It seems that the exaggerated postprandial lipemia in children and adolescents only concerns those with underlying lipid metabolic disorders.

The post-prandial lipemia curve is evaluated by the magnitude of the response to the stimulus (duration and/or extension) which corresponds to the time it takes to return to the baseline TG value, and by the amplitude that corresponds to the time at which the maximum value found occurs after lipid overload [15]. In normolipemic individuals at fasting, an increase is shown in the concentrations of lipoproteins rich in TG, in the form of an ascendant curve 2 h after eating, with its peak occurring at approximately the 4^th^ hour, and with return to baseline values close to the 6^th^ hour [16]. However, in individuals with dyslipidemia, the peak of lipoproteins rich in TG is observed later, between the 4^th^ and 6^th^ hours, with the return to baseline levels taking longer (after 8 hours) [17]. The same phenomenon occurs in individuals with insulin resistance [15]. In the present study, it was observed that adolescents with excess weight, irrespective of the presence of fasting hypertriglyceridemia, presented the TG peak in the fourth hour, and the eutrophic adolescents, independent of the fasting state, had an earlier TG peak in the second hour. However, considering the increase in TG concentrations (Δ T4- T0), no difference was noted between the two groups.

It was also observed that adolescents with central obesity presented significantly higher levels of TG at 4 hours, insulin and HOMA-IR, than adolescents without this comorbidity. Moreno *et al.*[14] found that adolescents with central obesity had higher TG levels post-prandially compared with those with a peripheral pattern of fat distribution. Another interesting finding in the present study was the correlation between waist circumference and TG at 4 hours (rather than fasting TG), which corroborates the study by Rie Oka *et* al. [17] that evaluated 1.505 men and 798 women aged 38-65 years and concluded that postprandial TG has a better relationship with waist circumference than fasting TG.

In the present study, it was also shown that only the adolescents with excess weight and fasting hypertriglyceridemia presented significant differences between the baseline and post-prandial TG values. In the postprandial state the persistent elevation of lipoproteins, rich in TG, can cause endothelial dysfunction, less availability of nitric oxide and increase of oxidative stress [18]. Couch *et al.*[19] studied the postprandial TG response to a fat load in children and their mothers from families with or without a family history of premature CHD (Columbia University Biomarkers Study) and found that a significantly greater postprandial TG response occurs in children with elevated fasting TG levels.

Nakajima et al. [20] evaluated 23 overweight to obese adult volunteers and demonstrated a stronger correlation between plasma TG and remnant lipoprotein triglyceride (RLP-TG) in the postprandial state than in the fasting state. This study suggests that non fasting TG measurements could replace direct measurement of remnant lipoproteins for the assessment of cardiovascular disease risk.

In this group of adolescents with excess weight and fasting hypertriglyceridemia, the post prandial levels increased significantly up to the fourth hour. This fact did not occur in the eutrophic adolescents with fasting hypertriglyceridemia. Humans rarely consume a single meal during the day [21]. Consumption of a subsequent meal causes a higher TG concentration than that which occurs after the first meal, even if the two meals are identical. As a result of this “second meal effect” the TG released can contain a significant amount of lipid from the previous one [21]. Subjects with obesity, diabetes mellitus and metabolic syndrome present postprandial hyperlipidemia [22]. One of the proposed mechanisms involves the competition between endogenous and exogenous TG-rich lipoproteins (TRLs) at different TG catabolic sites by overproduction of very low-density lipoproteins (VLDL) in the liver, in part due to hepatic insulin resistance [23]. Umpaichitra *et al. *[24] studied adolescents aged 10-19 years old with or without type 2 diabetes mellitus (obese and non-obese). They found that postprandial hyperlipidemia in response to a fat loading test is present in adolescents with type 2 diabetes mellitus who already have fasting hypertriglyceridemia.

In this study, no alteration was observed in the post-prandial levels of TC and LDL in the adolescents with excess weight and eutrophic adolescents. It has been well established that the ingestion of a meal rich in fats causes and increase in plasma triglycerides, whereas the concentrations of cholesterol are not significantly changed [25].

One of the difficulties in establishing and using the measurements of post-prandial lipemia in daily clinical practice is the lack of an easy to use methodological protocol for general use. In the pediatric population, there is no consensus as regards the type of food, quantity of fat ingested, time of collection after overload and normal values after lipid overload. The protocol used in the present study has the advantage of being sufficiently simple, both from the point of view of preparation and duration, using products to which there is easy access in any country. The “shake” offered contained 25 grams of fat, considering that a typical daily pattern includes about 3-4 meals and each meal can contain 20-40 g of fat [26]. In addition, the 4-hour duration protocol has been justified by previous studies [27,28] that have investigated postprandial lipemia and shown that the fourth hour after fat load test is the most representative time to measure the TG response. The Expert Panel Statement [26] suggests that a single TG measurement at 4 h after a fat loading test may represent a good estimation of the postprandial TG response.

The alterations resulting from the increase in lipoproteins do not depend only on their elevation (quantitative), but also on the qualitative characteristics of the diet (saturated, polyunsaturated and monounsaturated fats). Saturated fatty acids, with the exception of Stearic acid, increased the serum levels of all lipoproteins, particularly LDLc, since they reduce the synthesis and activity of the LDLc receptors by diminishing the expression of RNAm and membrane fluidity [29]. It has been suggested that the ingestion of saturated fat is the main dietary cause of elevation of plasma cholesterol [30]. A possible explanation for the absence of post-prandial elevation of LDLc and TC in the studied adolescents was the small quantity of saturated fatty acid used in the experiment (10.7% - 2.7 g/100 ml). In addition, the quantity of polyunsaturated fatty acid used (28.6% - 7.2 g/100 ml), may have influenced the LDLc and HDLc levels, because not only do polyunsaturated fats reduce LDLc, but they also reduce HDLc, thus inducing greater lipid oxidation [30]. Monounsaturated fats are as effect in the reduction of TC as are polyunsaturated fats, however, without causing lipid oxidation and reduction in HDL concentrations. In the shake offered to the studied adolescents, the largest proportion of fat (60.7% - 15.2 g/100 ml) was monounsaturated fatty acid.

A meal that contained up to 15 g of fat was associated with minimal (20%) increases in post prandial TG peak levels, whereas high-fat meals (e.g., 50 g), including those served in popular fast-food restaurants, increased triglyceride levels by at least 50% beyond fasting levels [31]. Approximately one out of five children with a BMI above the 95th percentile is hypertriglyceridemic, a rate that is 7-fold higher than for nonobese children of 6 to 10 years of age [31]. The genetic abnormalities of triglyceride metabolism that may be identified in childhood are rare and generally diagnosed soon after birth. More commonly identified are milder triglyceride level elevations (ie, 100 to 500 mg/dL) due to obesity, the major cause of pediatric hypertriglyceridemia [31]. Therefore, the prime target in counseling young children and their parents should focus on fat quality and quantity and then, in the early detection of underlying dyslipidemia [26].

The strengths of this study include its large sample size, measurements of fasting, 2 and 4 hours postprandial TG levels on the same day in each individual. The test drink studied here has been shown to be palatable and acceptable to adolescents, and it provides reproducible evaluation of post-challenge triglyceride profiles. In this study the nutrient intake (in terms of calories) was comparable to meals commonly consumed in typical fast-foods all over the world.

In conclusion, the behavior of lipoproteins in the post-prandial state in eutrophic adolescents and those with excess weight is similar. Thus, apparently the weight excess does not induce post prandial lipemic alterations.

## Competing interests

The authors declare that they have no competing interests.

## Authors’ contributions

The work presented here was carried out in collaboration between all authors. VS, SF and LFA defined the research theme. VS and LFA designed methods, analyzed the data, interpreted the results and wrote the paper. SF worked on associated data collection and their interpretation. All authors read and approved the final manuscript.

## References

[B1] Jackson KG Poppitt SD Minihane AM Postprandial lipemia and cardiovascular disease risk: Interrelationships between dietary, physiological and genetic determinants Atherosclerosis 2012 220 22 33 10.1016/j.atherosclerosis.2011.08.012 21955695

[B2] Alipour A Elte JW van Zaanen HC Rietveld AP Castro CM Novel aspects of postprandial lipemia in relation to atherosclerosis Atheroscler Suppl 2008 9 39 44 18595782 10.1016/j.atherosclerosissup.2008.05.007

[B3] Patsch JR Miesenbock G Hopferwieser T Relation of triglyceride metabolism and coronary artery disease. Studies in the postprandial state Arterioscler Thromb 1992 12 1336 1345 10.1161/01.ATV.12.11.1336 1420093

[B4] Lupattelli G Pasqualini L Siepi D Increased postprandial lipemia in patients with normolipemic peripheral arterial disease Am Heart J 2002 143 733 738 10.1067/mhj.2002.120302 11923813

[B5] Kolovou GD Daskalova DC Iraklianou SA Postprandial lipemia in hypertension J Am Coll Nutr 2003 22 80 87 12569118 10.1080/07315724.2003.10719279

[B6] Parks EJ Recent findings in the study of postprandial lipemia Curr Atheroscler Rep 2001 3 462 470 10.1007/s11883-001-0036-5 11602066

[B7] Zilversmit DB Atherogenesis: a postprandial phenomenon Circulation 1979 60 473 485 10.1161/01.CIR.60.3.473 222498

[B8] Glowinska B Urban M Koput A Galar M New atherosclerosis risk factors in obese, hypertensive and diabetic children and adolescents Atherosclerosis 2003 167 275 286 10.1016/S0021-9150(03)00003-0 12818410

[B9] De OM Onyango AW Borghi E Siyam A Nishida C Siekmann J Development of a WHO growth reference for school-aged children and adolescents Bull World Health Organ 2007 85 660 667 10.2471/BLT.07.043497 18026621 PMC2636412

[B10] Fernandez JR Redden DT Pietrobelli A Allison DB Waist circumference percentiles in nationally representative samples of African-American, European-American, and Mexican-American children and adolescents J Pediatr 2004 145 439 444 10.1016/j.jpeds.2004.06.044 15480363

[B11] Friedewald WT Levy RI Fredrickson DS Estimation of the concentration of low-density lipoprotein cholesterol in plasma, without use of the preparative ultracentrifuge Clin Chem 1972 18 499 502 4337382

[B12] Mehmet K Selim K Mustafa Kendirci M Emre A Cevat Y Homeostasis model assessment is more reliable than the fasting glucose/insulin ratio and quantitative insulin sensitivity check index for assessing insulin resistance among obese children and adolescents Pediatrics 2005 115 4 500 503 10.1542/peds.2004-1921 15741351

[B13] Moreno LA Quintela I Fleta J Postprandial triglyceridemia in obese and non-obese adolescents. Importance of body composition and fat distribution J Pediatr Endocrinol Metab 2001 14 193 202 11305798 10.1515/jpem.2001.14.2.193

[B14] Tiret L Gerdes C Murphy MJ Postprandial response to a fat tolerance test in young adults with a paternal history of premature coronary heart disease - the EARS II study (European Atherosclerosis Research Study) Eur J Clin Invest 2000 30 578 585 10.1046/j.1365-2362.2000.00674.x 10886297

[B15] de Ugarte MT Portal VL Dias AA Schaan BD Metabolic response to oral lipid overload in diabetes and impaired glucose tolerance Diabetes Res Clin Pract 2005 69 36 43 10.1016/j.diabres.2004.11.011 15955386

[B16] Maggi FM Raselli S Grigore L Redaelli L Fantappie S Catapano AL Lipoprotein remnants and endothelial dysfunction in the postprandial phase J Clin Endocrinol Metab 2004 89 2946 2950 10.1210/jc.2003-031977 15181082

[B17] Oka R Kobayashi J Miura K Difference between fasting and nonfasting triglyceridemia; the influence of waist circumference J Atheroscler Thromb 2009 16 633 640 10.5551/jat.406 19729868

[B18] Anderson RA Evans ML Ellis GR The relationships between post-prandial lipaemia, endothelial function and oxidative stress in healthy individuals and patients with type 2 diabetes Atherosclerosis 2001 154 475 483 10.1016/S0021-9150(00)00499-8 11166782

[B19] Couch SC Isasi CR Karmally W Predictors of postprandial triacylglycerol response in children: the Columbia University biomarkers study Am J Clin Nutr 2000 72 1119 1127 11063438 10.1093/ajcn/72.5.1119

[B20] Nakajima K Nakano T Moon HD The correlation between TG vs remnant lipoproteins in the fasting and postprandial plasma of 23 volunteers Clin Chim Acta 2009 404 124 127 10.1016/j.cca.2009.03.051 19345200 PMC3044434

[B21] Lambert JE Parks EJ Postprandial metabolism of meal triglyceride in humans Biochim Biophys Acta 2012 1821 721 726 10.1016/j.bbalip.2012.01.006 22281699 PMC3588585

[B22] van Wijk JP Halkes CJ Erkelens DW Castro CM Fasting and daylong triglycerides in obesity with and without type 2 diabetes Metabolism 2003 52 1043 1049 10.1016/S0026-0495(03)00106-9 12898471

[B23] Halkes CJ Van DH De Jaegere PP Postprandial increase of complement component 3 in normolipidemic patients with coronary artery disease: effects of expanded-dose simvastatin Arterioscler Thromb Vasc Biol 2001 21 1526 1530 10.1161/hq0901.095276 11557683

[B24] Umpaichitra V Banerji MA Castells S Postprandial hyperlipidemia after a fat loading test in minority adolescents with type 2 diabetes mellitus and obesity J Pediatr Endocrinol Metab 2004 17 853 864 15270403 10.1515/jpem.2004.17.6.853

[B25] Axelsen M Smith U Eriksson JW Taskinen MR Jansson PA Postprandial hypertriglyceridemia and insulin resistance in normoglycemic first-degree relatives of patients with type 2 diabetes Ann Intern Med 1999 131 27 31 10391812 10.7326/0003-4819-131-1-199907060-00006

[B26] Kolovou GD Mikhailidis DP Kovar J Assessment and clinical relevance of non-fasting and postprandial triglycerides: an expert panel statement Curr Vasc Pharmacol 2011 9 258 270 10.2174/157016111795495549 21314632

[B27] Nordestgaard BG Benn M Schnohr P Tybjaerg-Hansen A Non-fasting triglycerides and risk of for myocardial infarction and death among women and men Ugeskr Laeger 2007 169 3865 3868 18031660

[B28] Weiss EP Fields DA Mittendorfer B Haverkort MA Klein S Reproducibility of postprandial lipemia tests and validity of an abbreviated 4-hour test Metabolism 2008 57 1479 1485 10.1016/j.metabol.2008.05.020 18803956 PMC2585379

[B29] Schaefer EJ Lipoproteins, nutrition, and heart disease Am J Clin Nutr 2002 75 191 212 11815309 10.1093/ajcn/75.2.191

[B30] Signori LU Plentz RD Irigoyen MC Schaan BD The role of post-prandial lipids in atherogenesis: particularities of diabetes mellitus Arq Bras Endocrinol Metabol 2007 51 222 231 10.1590/S0004-27302007000200011 17505629

[B31] Miller M Stone NJ Ballantyne C Triglycerides and cardiovascular disease: a scientific statement from the American heart association Circulation 2011 123 2292 2333 10.1161/CIR.0b013e3182160726 21502576

